# Do clients train therapists to become eclectic and use the common factors? A qualitative study listening to experienced psychotherapists

**DOI:** 10.1186/s40359-022-00886-6

**Published:** 2022-07-27

**Authors:** Douglas Behan

**Affiliations:** School of Social Work, Rutgers, The State University of New Jersey, 390 George St. 3rd Floor, New Brunswick, NJ 08901 USA

**Keywords:** Common factors, Therapeutic relationship, Eclectic

## Abstract

**Background:**

Psychotherapists must choose from an overwhelming number of theoretical models and empirically supported treatments to guide their work. Meta-analytic studies show there is comparable efficacy among the choices, making the decision about which approach to use difficult. Research indicates there are pantheoretical elements found in all effective models, called the common factors, which can offer psychotherapists a focusing point to maximize their effectiveness regardless of their chosen approach. Most psychotherapists begin practicing from a traditional theoretical orientation, but then their approach evolves over time toward an unintentional eclecticism, derived primarily from their practice experience with clients.

**Methods:**

This exploratory qualitative study conducted in-depth interviews with six experienced clinical social workers about their evolution as psychotherapists and what they believe creates change in psychotherapy. The interviews were conducted using standardized prompts and then coded and analyzed utilizing thematic analysis based on a six-phase framework.

**Results:**

The analysis suggests the psychotherapists had evolved to conducting therapy via an implicit and unique approach based on an unintentional heavy use of common factors. Five prominent themes emerged as central components of change in psychotherapy: the therapeutic relationship as a primary change agent, the importance of the therapist genuineness, the need to acknowledge and act upon a poor therapist—client match, the client bearing the primary responsibility for change, and the therapists’ development of unintended eclecticism in response to client interactions.

**Conclusions:**

In practice, most psychotherapists start practicing from a traditional theoretical orientation only to find their approach evolves over time toward an informal eclecticism featuring common factors. This common factors-based eclecticism emerges primarily from practice experience with clients. These findings suggest an avenue for further inquiry—if psychotherapists are going to gradually evolve in an unplanned eclectic direction guided by their client interactions, are they also concurrently and inherently drawn to the common factors? If the answer proves to be yes, what are the implications for early training? Should the gradual emphasis toward common factors be supplanted with a more intentional and efficient focus on them in the training of students and early career clinicians?

## Background

In 1952, the legitimacy of psychotherapy as an effective healing modality was called into question. Hans Eysenck [1] conducted a review of the existing research on psychotherapy outcomes and came to the unsettling finding that psychotherapy failed to facilitate recovery. This challenge to the effectiveness of psychotherapy has since been thoroughly addressed by research that has repeatedly demonstrated its effectiveness [2, 3]. Thousands of outcome studies have shown a variety of psychotherapy models to be effective at treating a range of disorders—achieving an overall success rate of 67% compared to a 33% improvement rate in untreated individuals over the same timeframe [4]. However, the subsequent question of *how* psychotherapy is effective has not been so clearly answered. Over 400 different psychotherapy models exist—each with a distinct perspective on how to conceptualize and treat mental illnesses [5, 6]. Practitioners confronted with this overwhelming array of models will naturally want to know, “Which psychotherapy model is best? What does the research say?” Numerous models have been rigorously tested and can wear the badge of an empirically supported treatment, but how does a clinician choose among them? While many treatment approaches can demonstrate efficacy, no theoretical orientation or model has been shown to be superior to the others in treating a range of conditions. There is evidence that some models that emphasize exposure to stressors are marginally more effective than other models in treating specific conditions such as phobias [2, 7] but based on meta-analytic reviews of outcome studies [7], no model or theoretical orientation has emerged as superior to the others in treating the broad array of mental disorders encountered by psychotherapists. Hundreds of models have been created, thousands of studies have been conducted, and no clear winner has emerged.

One answer to this dilemma posits that it is erroneous to assume that a model and its specific techniques are the active ingredients bringing about change in psychotherapy [8]. Perhaps focusing on the model as the primary change agent may simply be the wrong paradigm. The common factors perspective builds from this premise and asserts that psychotherapy is effective because of elements that are common among seemingly disparate models [7, 9, 10]. It is a compelling proposition that common factors found in all psychotherapy models function as a skeleton key to unlock the changes needed for improvement. If we can better identify these common factors and purposefully and strategically deploy them, then therapy outcomes should improve. Exploration of the common factors has received sustained attention over the years by a comparatively small but dedicated group of researchers [for summaries see, 11, 12]; however, the overwhelming focus in research and practice has been on the continued use of specific therapy techniques and models [10, 13].

Many have called for integrating common factors into integrative practice [14–16]; however, most beginning clinicians are still traditionally guided to adopt a single theoretical orientation, often the one endorsed by their training institution, which they subsequently use to guide their psychotherapeutic formulations and interventions [17]. Some students may receive exposure to the common factors literature, particularly if they are attending one of the few programs with a defined integrative psychotherapy curriculum [14, 18], but generally exposure to common factors theory and research, if addressed at all, is embedded within a program that endorses using a single therapeutic orientation.

One barrier to the formalized adoption of the common factors into practice is that no definitive list of the common factors exists, though attempts have been made to group them. In a review of the common factors literature, Grencavage and Norcross [19] identified 89 common factors, sorting them into five categories (a) the therapeutic relationship, (b) therapist qualities, (c) client characteristics, (d) change processes, and (e) treatment structure. The single most identified common factor in the literature is the therapeutic relationship [20]. Development of a therapeutic relationship based on empathy, warmth, and a working alliance is the most prominent and accepted common factor [21] as it is seen as central to the successful delivery of any psychotherapy model [22, 23]. However, with the exception of the therapeutic relationship, which holds consensus as a common factor, more research is needed to refine and codify the common factors to support training programs [14]. Others have argued that the qualities that the client brings to treatment have the most impact on psychotherapy outcomes, such as their support system, motivation and involvement in treatment, resilience, and self-healing abilities [7, 24, 25].

Adopting and using a single therapeutic model approach, although often espoused as good practice, does not reflect the reality for many psychotherapy clinicians in practice because most clinicians develop in an integrative or eclectic direction over the course of their professional career ([26, 27]. When confronted with the diverse challenges of actual practice, a single theoretical model proves to be an insufficient guide, leading to the unintended but necessary progression toward an integrative practice [28, 29]. Qualitative research by Rihacek and Danelova [27] examined the career progression of 22 integrative psychotherapists and found their career progressed along three stages: (1) adherence—where they initially followed a single theoretical orientation, (2) destabilization—where the demands of practice exposed the limits of their adopted orientation. Therapists in this stage found their home orientation was insufficient at meeting the array of issues their clients presented with. Also, disagreements emerged with the epistemological foundations of their model, as it did not adequately explain the dynamics encountered with many clients. This dissonance with their model led them to (3) consolidation—a stage characterized by a synthesis of theory and techniques from disparate theoretical orientations into a personal theory either at an implicit or explicit level. This stage involved an incorporation of outside influences into their approach, albeit in a somewhat haphazard or fragmented manner.

A key finding of Rihacek and Danelova’s research is that this integrative approach developed by clinicians is generally *unintended*. Clinicians are not embarking on a chosen path using existing integrative models, instead they are moving toward an unsystematic eclecticism without specific guidelines [29]. This unsystematic and idiosyncratic career evolution is a troubling finding given the current widespread call for evidenced-based practice. The scale of this issue is large; beginning in the 1990’s, surveys have found eclectic/integrative to be the largest reported theoretical orientation by psychotherapists [5] or second largest orientation (following cognitive-behavioral) [30]. However, even though vast numbers of clinicians are practicing in an eclectic way, little research has been conducted to examine how they typically move toward more eclecticism/ integration [31]. It seems eclectic practice is common, arrived at without specific guidance or intention, and not well examined by the research community.

So why are so many therapists departing from models they were trained in and evolving into an unplanned eclectic/integrative orientation? One reason may be that the current evidence base has primarily examined pure-form models giving students and clinicians the impression that an evidence-supported pure form approach is the gold standard [32]. Models used in research studies are often limited in scope to target specific behaviors and often delivered with the use of a manual [33]. This circumscribed approach yields a good research design by reducing study variables, but it is not easily replicated in real-world practice [34]. The evidenced-based treatments from studies are simply not finding their way into routine practice [35, 36].

A second possibility is that when clinicians encounter the destabilization stage described by Rihacek and Danelova [27], they are not turning to current research to address the gaps in knowledge, theory, or technique they are experiencing. Practitioners typically do not have access to academic journals where the latest research findings are published, and they may be overwhelmed by the sheer number of books, models, techniques, and research findings all claiming to be the best approach [37]. Access to qualified training and insufficient time and financial resources have also been found to be impediments in psychotherapists adopting new approaches [38]. Instead, clinicians are generally moving beyond the perceived limits of their initial theoretical approach and building their own personal theory to understand and treat mental disorders [39, 40].

Therapists have identified working with patients as the most potent influence on their practice—above theory, research findings, supervision, or professional training [41]. The therapist’s “personal theoretical orientation” emerges from his or her own “research”—the intense work they are doing day-in, day-out conducting psychotherapy. Evidence-based practice acknowledges the value of this hard-won knowledge, generally calling it "practice wisdom", and gives it standing along with theory and research in framing good practice [42]. The demands of addressing the myriad of problems encountered with diverse clients pushes therapists away from a purist mentality and into a practical approach of finding out what works for each client [43]. A survey by Thoma and Cecero [44] found that even therapists espousing to be pure-form clinicians (following cognitive-behavioral, psychodynamic, or humanistic orientations) reported using more techniques from outside their orientations than from within their orientation.

The research community cannot hope to fully support clinicians in dealing with all they encounter in the therapy room. It is highly improbable that every type of therapy will be tested against every disorder to produce an affirmed best practice [45]. Consequently, each therapist is creating their own path. In a qualitative study of 100 therapists, Randstad and Skovholt [46] found that experienced therapists reported professional growth that is primarily self-directed and not limited to traditional sources of professional learning. Their approach to therapy was enriched through other domains of learning in their life, such as literature, philosophy, movies, and their own life experiences. A professional evolution occurred allowing the therapists to rely on internal expertise to guide their practice—whereas less-experienced clinicians sought direction from external expertise.

It may be easy to frown upon therapists developing their own unique theoretical orientation as unscientific and unsupported by research, but in effect, therapists are the ultimate ethnographers, spending their career observing the challenges brought to them by clients and together testing curative interventions “in the field”—to bring about positive change. Perhaps we should be listening more deeply to these “researchers” to learn what they know about change factors in the therapy process. Their tacit knowledge is derived from praxis, as they continually meet the challenge of helping each unique client presenting with a unique constellation of symptoms and problems.

There are calls for more research to discern what experienced therapists believe are the factors that elicit change in psychotherapy [30, 45]. This exploratory study is a step towards answering this call. This study focused on tapping the implicit procedural knowledge of experienced therapists, seeking their insights as to what factors create change in psychotherapy through an intensive interview process. The aim of this study is to (a) deeply listen to a group of experienced psychotherapists describing what they view as the primary change factors in psychotherapy, (b) understand if and how their reports relate to the use of common factors, and (c) understand how their practice approach has evolved over the course of their career.

## Methods

### Participants

#### Eligibility

Sampling criteria for this project were as follows: study participants needed to be licensed as a clinical social worker, with a master’s degree or above, with a minimum of 10 years of experience, who were currently conducting psychotherapy with adults. The 10 years of experience may have been obtained in various practice settings, but the participants were required to currently be working as a private practice therapist where they have the autonomy to conduct psychotherapy using a treatment approach of their own choosing. Participants working in a setting where the treatment approach may be directed by an agency or supervisor were excluded. Participants working with children were excluded because psychotherapeutic approaches with children can differ significantly than those used with adults, resulting in potentially different mechanisms of change.

#### Recruitment

For this qualitative pilot study, a sample size of six experienced psychotherapists was sought with the goal of generating in-depth case studies that would help distill areas of inquiry for future larger-scale studies of experienced clinicians. A small sample size was chosen to offer the richness and depth of a case-oriented analysis. Convenience sampling was used via a publicly available database of licensed clinical social workers in the US state where the study was conducted. Clinical social workers are the most abundant mental health providers in the US, offering a homogeneous group of experienced psychotherapists for the pilot study. The database was sorted by license type (clinical or non-clinical), date of issue, and county of residence, which allowed clinical social workers holding a license for 10 or more years in three counties to be contacted. A mass email with the subject line “Experienced psychotherapists sought for research study” was sent. The email recipients were invited to contact the researcher if they were interested in participating in a study “examining the factors of change in psychotherapy.” The recruitment email informed recipients that participation would require 90 min of their time and that the interview could be conducted in their private practice office.

#### Participants

284 emails were sent, and 48 individuals responded*.* Participants were contacted in the order they responded to the recruitment email and the first six that were available and met the study criteria were selected to be interviewed. Six individuals between the ages of 47–77 were interviewed, with a mean age of 60.3 (*SD* = 11.99). There were five women and one man. Five of the individuals held a master’s degree in social work and one held a doctorate. The average number of years the individuals had practiced as a licensed clinical social worker was 27.83 (*SD* = 10.85). The average number of years in private practice was 20.16 (*SD* = 9.76) and the average number of clients seen per week by the group was 17.16 (*SD* = 10.85). Participants were asked to indicate their theoretical orientation from the following choices: (a) cognitive-behavioral, (b) Eclectic/Integrative, (c) Humanistic, (d) Psychodynamic, (e) Systems-theory, (f) Other (please specify), and (g) I don’t have one. The participants reported varied primary theoretical orientations, with two individuals identifying as psychodynamic, one as gestalt, two as cognitive-behavioral, and one as systems-theory.

#### Data collection

A thirteen question semi-structured interview guide was developed that focused on eliciting thoughts about the change factors in psychotherapy (see Appendix). Questions were open ended to encourage in-depth responses, such as: Tell me about your approach to conducting psychotherapy. Do you conduct therapy differently at this point in your career than when you started? and, what makes you effective? Qualitative interviews were used to obtain various perspectives on the research questions regarding change factors in psychotherapy. After informed consent was ensured, each interview was conducted by the principal investigator in the participant’s private practice office, with each interview lasting 90 min. All interviews were audio recorded and later transcribed verbatim for analysis. Files were dated and given a unique identifier and archived in a secure network location. Participants were informed that their responses would be kept confidential and that the study design was to aggregate and anonymize all the study interviews and to investigate themes in the data rather than to focus the analysis specifically on any one respondent.

#### Data analysis

Data were analyzed by the principal investigator utilizing the six-phase theoretical framework for thematic analysis developed by Braun & Clarke [47]: (1) interviews were transcribed and then repeatedly read to gain familiarity with the breadth and depth of the content. Notes and memos were created to identify potential codes and areas for analysis; (2) the interview transcripts and audio recordings were then imported into the coding software NVivo (ver. 12) which was used to generate an initial set of codes. The software assisted in developing coding schemes and managing large blocks of text. The initial set of codes were then analyzed for patterns of meaning, leading to further refinement of codes and subcodes and the subsequent reduction of the code set; (3) the remaining codes were then sorted into eight candidate themes reflecting broader patterns of meaning; (4) the candidate themes were reviewed and further consolidated into five superordinate themes that best represented the data set; (5) a reliability check was conducted in which an experienced qualitative researcher not involved in the study coded a sample of 60 data samples into the previously determined superordinate themes. A 93% congruence rate was achieved with the initial coding. Of the four codes lacking consensus, two were recoded and two were deemed applicable to more than one theme; (6) the themes were then further analyzed for their connection to the research questions and the aspects of the data set that each theme captured leading to a more in-depth understanding of the “story” told by each theme. Sub themes were developed to best capture the nuances of meaning within each theme; (7) the report was produced based on the refined themes.

## Results

The concept of “common factors” was purposefully not included in the interview questions and was not raised by the interviewer nor was the term specifically mentioned by any of the participants. However, through the application of thematic analysis, several of the common factors of effective psychotherapy were found to be represented in the data. The five common factors most strongly represented in the data set were prominently discussed by each respondent. They will be described in further detail. Additional common factors emerged as themes but with less intense representation, including: (a) the need for the therapist and client to develop a shared conceptual frame about the presenting problem; (b) the importance of therapist traits such as confidence and warmth; (c) establishing role clarity and expectations for the work of therapy; and (d) building hope for improvement.

The Five Most Prominent Themes in the Data Set:

1. The therapeutic relationship is a primary change agent.
2. The therapist must be genuine.
3. Take action when the client—therapist match is not working.
4. The client is the person most responsible for change.
5. Over time, psychotherapy practice evolved into an unintentional responsive eclecticism.

### Theme one: the therapeutic relationship is a primary change agent. “*Understood in a really deep, subtle level”*

When asked “what creates change in psychotherapy”, each respondent in this study described the therapeutic relationship as being the foundational component of change in the therapy process. The therapist—client relationship had the most robust representation in the data and was described by the participants as something significantly more than simply establishing a platform to support delivery of therapeutic interventions; the therapeutic relationship was seen as an intervention in itself. One respondent described the role of the therapeutic relationship as:

It’s a corrective emotional experience, there's some new kind of connection, some way of being heard, some way of being listened to, some way of being responded to that's *different*. That's fundamentally different than what [clients] had before. They’re understood in a really deep and subtle level.

Regardless of their original theoretical orientation, each respondent emphasized that a strong and reciprocal therapeutic relationship with the client is a curative element that is a foundation for the psychotherapy process to work. Carly (all participants in the study have been assigned a pseudonym), a therapist with decades of experience, elevates the therapeutic relationship to a level of primacy, “I think it is 90% personal relating—on a personal and professional level.” The descriptions of the centrality of the relationship were not described as part of a specific model or interventional approach but seemed to be used in an analogous way to describe the therapy process itself. Another experienced therapist described the pantheoretical role of the relationship:

The “relationship,” I feel like it's so overused, but it's so key for clients to be able to feel comfortable with me, or whoever they pick, and to have this kind of rapport or working alliance, there's all kinds of names for it, but it's *real*. I think it is really powerful when people can absolutely be themselves and talk about things they would never imagine talking about.

One respondent described how her clients benefitted from being in an ongoing relationship with her—more so than any interventions she used with them. She succinctly describes how she sees the essential importance of the relationship, elevating it above her clinical knowledge, theoretical orientation, and therapeutic skills:

It's being a person with another person. You can be a lot of things, you can know a lot of stuff, but you don't really have to know a lot of stuff, you just only have to be a human being with another human being. So, however that adds up, that's probably what I do best.

### Theme two: the therapist must be genuine. *“You have to be genuine; they want to see you as genuine”*

A concept that received considerable representation in the data was the need for the client to perceive the therapist as genuine—being a person first, a therapist second. Achieving genuineness was seen as difficult, with many barriers to its manifestation. One experienced respondent stated, “I’ve learned that the more vulnerable you can be, the more effective you can be too. *If* you can allow your own vulnerability.” Each respondent described great difficulty being fully genuine with clients early in their career—they did not feel entirely natural and confident in their role. Betty stated, “When I first started out, I was thinking much more about the books, much more about the theory. I'm much more comfortable now, I'm much more real.” As their career evolved, they became freer to be themselves in their work with clients, which they identified as a significant improvement to the therapy they provided. Abby stated:

Being genuine and being present. Letting yourself be hit again and again. Being vulnerable to what comes. Being open to it. Being that kind of martial artist that can catch a thing and hold it or put it somewhere instead of a feeling assaulted by it.

The participants found a way to offer the client their authentic self while still maintaining their therapeutic stance. Darren stated, “I think by the time you have been doing this for 20—25 years it's just easier to be yourself, and just have good boundaries.”

To be genuine with a client poses a constant challenge of vulnerability to therapists, as one study participant put it, “It's hard to be real. Even in my own marriage. Really, be real with somebody? That's *hard*.” However, if the therapist hides behind a professional persona or technical expertise, the client notices this and the work together is compromised. Edna, a clinician who spent many years working with multi-problem clients in mental health clinics states, “You have to be genuine; they want to see you as genuine”. Another participant contributed, “It’s really being Carl Rogers in that sense, really being genuine. Clients know immediately if you’re not being genuine. Everybody involved knows that. And I've made my mistakes.” The emphasis on genuineness by the participants was based on the belief that clients need to see that the therapist is truly present with them *as a person*, something that cannot be conveyed through skills or technical expertise. It calls for vulnerability on the part of the therapist, a stepping out of the professional persona. The client’s need for genuineness is succinctly captured by a participant who stated, “You have to feel your therapist respects you and likes you in a certain way. *And it has to be true*.” The idea of being genuine or “real” was elevated above other elements of the therapeutic relationship, such as warmth or empathy. Another, a seasoned therapist, stated:

I think there are so many common vernacular understandings of empathy. It’s like, "put yourself in somebody else's shoes" but I think you'll still be *yourself* in their shoes [laughs]. I mean that. I think that being real is more important than empathy actually.

### Theme three: take action when the client—therapist match is not working.: ***“I know who I work well with”***

Participants described the therapeutic relationship as so foundational to conducting effective therapy that they are resolute in ending treatment with a client when the therapeutic relationship is not working out. This yes/no decision on the viability of the therapy was reported to be made quickly and decisively, often within the first three sessions. One respondent stated, “If there’s a good fit, I think the client will trust and try what I suggest. I think they will give me credibility, and if there isn’t, there is absolutely no point in doing the work to begin with.” Little pretense was reported over trying to form a relationship if it was deemed unlikely to work out. Dana, a therapist who has been in private practice for over twenty years, states:

I know who I work well with at this point in time, but I didn't know that early on in my career. I know who I'm good with, and who I definitely don't work with well. And who is not likely to “click” well with me.

The decisiveness in determining the viability of the therapeutic relationship is not based on blaming the client or the therapist for not working hard enough; the decision that the therapy is not worth pursuing is based on a fundamental acceptance that not every pair of people can work well together. Dana stated:

It is just hard to know who you connect with. With some clients, there's a much better working connection and some it's immediately tenuous. Maybe there is somebody else better for them? It's a huge piece of the puzzle—just the natural chemistry that happens between two people, good or bad, for whatever reason.

This acceptance that the relationship is unlikely to support effective therapy, or that therapy is not what the client currently needs, is openly conveyed to the client, as one respondent describes:

One of the considerations is should the client go to someone else if there's nothing I can offer them? Do they need a fresh perspective, from a different angle? Sometimes it may be that they need someone else who’s different or just something else. If they're not ready to make the change even though they have the insight, they should go live for a while [leave therapy] and when they are ready, or something changes, they can come back.

For the relationship to work, both therapist and client must feel connected. Sometimes, the lack of connection may only be felt on the client’s part. The therapist needs to be open and resilient enough to accept that all clients will not connect with them. Put into action, the therapist gives permission to the client to broach the topic and possibly end the therapy. One respondent described her approach on raising this topic:

This is one thing I always say to clients, “I'm never going to be offended if you say this [therapeutic relationship] is not the right fit for you. This is perfectly fine. It's not just something I say. It’s the truth, and I will not be offended if you feel it is not a good fit.”

A distinction should be made that ending treatment was considered the right course of action when, early on, the initial therapeutic relationship was not seen as viable, however, participants did not indicate that termination should be considered when ruptures occur in already successfully formed relationships. Most participants viewed this circumstance differently and advocated repairing the rupture as the best course of action.

### Theme four: the client is the person most responsible for change: *“I’m just the vehicle, the client is the driver”*

The participants in this study strongly and repeatedly supported the idea that the client has the most influence over the therapy outcome, more so than the therapist or the therapeutic modality. Each of the participants made statements supporting this position, such as, “I’m not there to do the work; I’m just there to guide,” “I think we act as catalysts more than solve problems,” and “I'm just the vehicle. The client is the driver. They are in control.” The client’s role in the change process is seen as happening both within and outside the therapy sessions, with some emphasizing that change mostly occurs outside the therapy room, “the work of therapy happens between sessions,” and “the people who really do well and thrive, are working. They are thinking in between sessions, they're coming in with what they thought about.” This belief that clients are primarily responsible for change requires the therapist to socialize clients into a role where they are expected to work. To achieve this, participants reporting using statements such as:

I'm walking next to you; I'm not carrying you and I'm also not choosing your path. I'll walk next to you, point out for you, look out for traps you might fall in, but you are in control. I'm just walking and being present with you.

I'm not going to sprinkle magic dust on you and all your problems will go away. We can work to … identify things and come up with a plan perhaps. But you have to do the plan. Otherwise, I can't offer you something more.

The conviction that clients have the key role in the change process appears to develop from experience over time rather than it being part of their theoretical orientation. One respondent discussed how she came to understand this as her career evolved and reflected upon how beginning therapists struggle with assuming too much responsibility for change:

They [beginning therapists] are not responsible for everything that happens. They're not the one in charge, really. They feel such a burden of responsibility. First of all, it's very responsible people who become therapists, but you don't have a lot of control over very many aspects of this process. There is a lot that influences clients, not just you.

Another respondent acknowledges that the actual source of change is often unknown and humbly accepts that it could be based on something completely outside of the therapy purview:

Clients always say their improvement is because of some other reason besides therapy. They often say, "I’m feeling better. I'm not sure what it is. Maybe it’s the fact that I'm now taking vitamin D or something". And that is entirely possible, you know [laughs].

### Theme five: over time, psychotherapy practice evolved into an unintentional responsive eclecticism.: *“I am much more myself”*

The study participants each reported adopting a theoretical orientation at the outset of their career that has since been supplemented by experience, supervision, continuing education, and reading professional literature. When asked to discuss their views on what creates change in psychotherapy, participants generally did not cite tenets from their identified theoretical orientation. Instead, they brought up seminal experiences from their work with clients. They described successes and mistakes that have since shaped how they conduct psychotherapy. They clearly privileged learning from clients over all other sources of learning. None of the participants identified as following an eclectic or integrative model, however each described adding techniques and guiding concepts from outside of their “home” orientation over the course of their careers in order to respond to unique client situations. One respondent was more conscious about utilizing ideas from varied theoretical orientations:

Early on, everyone would say, "you can't say eclectic". They got upset because it sounds like you have no foundation. So, you were really pushed to pick something and dig in, which can be useful, but I think eclectic is good if you have some decent exposure. You are never going to get five theories down the way you can get one. But if you get some reasonable exposure to several, you can pick and choose.

The study participants followed the “pick and choose” approach. All exhibited a notable level of confidence in conducting therapy outside the dictates of a specific model; they used what made intuitive sense to them and adapted their approach to what they perceived each unique client required. The study participants described a transformation from being a rule-conscious and anxious clinician at the beginning stages of their career into self-assured and instinctive clinicians—earned via years of experience. They described a marked increase in trusting their therapeutic instincts—a certain “knowing” what they are doing makes sense, and a decreased need to seek external validation that their approach with a client is the “right’ one. One respondent stated, “I would say that my approach is open-ended and that it is certainly not manualized. When it feels right—when my felt sense tells me that I need to intervene in some way that's more active, I do.” Another reported a similar intuitive approach to her work, “I'm more honest now. I'm more skilled, for sure, but I'm more *seasoned* rather than skilled. I can use the skills in a more human way—instead of the work being about a book that I just read.” One respondent related a story from when she was a beginning therapist that made a strong impression on her. She asked a senior trainer about how he had changed over his career as a psychotherapist and he simply replied, “I am much more myself.” As a senior therapist herself, only now does she understand and relate to what this means.

## Discussion

The predominant research approach used to study psychotherapy outcomes has been based on the medical model in which there is a patient, who presents with some form of pathology, that is treated with an intervention (e.g., medication or surgical procedure), that is delivered by an expert (e.g., physician) [7]. This approach attempts to isolate and study the intervention as the curative factor. The common factors approach reverses this model, instead identifying the therapist and client as more potent change factors than the specific interventions used in therapy. In recognition of this, there are calls to shift more attention within psychotherapy outcome research toward therapists in order to better understand their perceptions of the therapy process [48, 49]. This study focused on the implicit procedural knowledge of experienced therapists, seeking their insights on the most salient change factors in their work.

A variety of theories and skills from across the major theoretical models were described in the interviews which demonstrated a level of eclecticism typically found in experienced psychotherapists [28]. The therapists interviewed each reported adopting a primary psychotherapy theoretical orientation at the onset of their career (i.e., psychodynamic, cognitive-behavioral, gestalt, and systemic) and currently view their work as still primarily guided by that model. However, when describing their current approach to conducting psychotherapy, they reported using concepts and skills from varied orientations and a ready willingness to deviate from their original model and “do what works” based on the needs of each case. Therapists in the study reported their therapeutic approach evolved significantly from their early years when they had less confidence and felt obligated to adhere to their theoretical orientation. They subsequently evolved a non-pure form of their original orientation that was more intuitive and that employed a variety of concepts and skills from disparate models. Working from an experienced derived personal therapeutic approach has been shown to occur in experienced therapists [40]. A study by Romaioli and Faccio [29] found therapists develop an “informal eclecticism” when their theoretical model proves inadequate. The experienced therapists in this study clearly fit the pattern of consolidating a unique yet implicit approach to their work. They were not avowed eclectics or integrative therapists, instead their approach evolved unintentionally—driven by doing what works with their clients rather than by what a book or model recommends.

With the end of the formal training of their early career, the respondent’s primary source of learning subsequently came from their reciprocal interactions with their clients. Clients became their primary teachers—not books, continuing education courses, or research. The utilization of research to guide practice was not mentioned by the clinicians, which is consistent with research findings that most clinicians do not directly use research to guide their practice [50–52].

When asked to illustrate change factors in psychotherapy, the experienced therapists in this study repeatedly referenced meaningful exchanges with clients that helped them evolve and refine their individualized approach to practice. They were “taught” by their clients that, to be effective, they must expand and deviate from their primary model to meet their client’s needs. This evolution of approach is not by design—it occurs in the crucible of the therapy space and is reinforced by the client discouraging rigidity and rewarding adaptability in approach. The study subjects learned these lessons intuitively from their clients as their careers progressed. Further exploration of the possibility that therapists privilege learning from clients over theory and research is warranted. If clients are the primary teachers, questions arise such as, what are clients teaching therapists? Are there predictable lessons that emerge over the course of the hours, weeks, and years conducting therapy? Are these lessons different from a therapist’s early formal training?

One of the questions of this study was to find out if experience leads therapists to emphasize common factors of psychotherapy in their work over more model specific interventions. The answer with this sample appears to be a clear “yes.” Studies have found that clients value the presence of the common factors over technical expertise when choosing a therapist [52, 53]. If these common factors are desired by clients, then hopefully therapists are taking note. According to the interviews collected, the therapists in this study did implicitly identify several of the common factors as key drivers of change in the psychotherapy they are conducting. Several common factors were strongly represented in the coded interviews, with the most prominent being the therapeutic relationship, client motivation, and therapist genuineness. Listening to clients appears to have fostered a greater reliance on the common factors in this qualitative study.

The gravitation toward the common factors was not by design however, as if the therapists were consciously deploying the common factors. Instead, it appears to be a natural evolution, driven by years of trial and error with clients, and learning how to increase effectiveness by doing what clients want and need. This confluence of common factors and the therapist’s naturally evolving implicit approach to their work warrants further attention with larger samples. If therapists are not sticking to pure models, not using research to inform their practice, and unintentionally evolving to use certain common factors based on practice experience, how can the research community best direct its efforts to impact psychotherapy practice? Perhaps education on the common factors should be a core curriculum in training programs instead of the pure form approach which still dominates.

The three common factors with the strongest representation in the data were the primacy of the therapeutic relationship, the need for genuineness by the therapist, and the importance of client motivation. The impact of a strong therapeutic relationship on positive psychotherapy outcomes has strong support in the literature and has the most empirical support of all the common factors [22, 23]. The therapists in this study clearly found a strong therapeutic relationship to be essential for therapy to be productive. So much so, that when they believed the relationship was not likely to achieve the level of cohesion required for effective work, they were quick to recommend ending treatment and refer clients elsewhere, or to recommend stopping treatment altogether. Most described making this determination in the first 1–3 sessions. This decisiveness appears to have developed later in their careers, after years of seeing the central role of the relationship and the futility of trying to force a strong connection when one is unlikely to develop. Their descriptions did not typically ascribe blame for the lack of connection but were based on acceptance that not every pair of people will work well together. A larger sample comparing the willingness level of early and late career therapists to end treatment due to an insufficient therapeutic relationship appears warranted.

The importance of therapeutic genuineness with clients was prominently represented throughout the interviews. The consistency of this finding across the six participants was surprising. Described in different ways, the study participants believed to achieve the person-to-person connection that makes therapy effective, it is vital to avoid artifice or appearing to be simply playing the role of a therapist in the eyes of the client. Given the therapists in this study had an average of 28 years of experience, this finding may be connected to the implicit and unique therapeutic approach found in experienced clinicians. Most described how theory-bound and nervous they were in the beginning years of their practice and how helpful and refreshing it is to now be themselves with clients. Based on this finding, helping clinicians in the beginning of their careers overcome nervousness and rigid adherence to theory so they can adopt a more genuine stance may improve outcomes, and warrants further study.

Some have suggested the client is the single most salient change factor in therapy [7, 24, 25, 54]. The calls for more research on the client’s role in bringing about change are further supported by the findings in this study [55]. The experienced therapists in this study had strong convictions about the importance of the client doing most of the work in therapy. They no longer harbored the self-blame they once experienced earlier in their careers when clients lacked motivation in therapy. Their need to “work harder” to make therapy successful was replaced with a calm acceptance that clients are often not ready or willing to do the work required to change. It appears experience has yielded them patience and acceptance in this regard. It has also empowered them to be more direct in deputizing clients to work on their own behalf when structuring the treatment process with them.

## Limitations

Limitations of this pilot study include the sample size of six participants. The findings provoke further research questions and are not generalizable. Although themes appeared consistent across the sample, a larger sample with varied demographics may have yielded other themes. Member checking was not included in the study design. There are transferability limitations. The sample was not diverse in terms of race or gender, with all participants being White and with one male respondent. All the participants were clinical social workers, results may be different with other mental health professionals. A larger quantitative study exploring the questions raised by this study’s findings should include a mix of mental health professionals to see if there are differences potentially attributed to professional training and approach. The participants were not geographically diverse, as all came from the same US state and worked in suburban or urban settings. Because the subjects were in private practice and volunteered for this study, they may have qualities that are not representative of other therapists such as a greater degree of autonomy and a preexisting interest in the change factors of psychotherapy. They also may have greater latitude to adapt their approach than clinicians in agency settings.

The data collected in this study are based on participants’ conscious thoughts about their psychotherapy practice that they were willing to openly share with the interviewer. The participants may not have been conscious of other relevant factors. Clinical success and their career evolution were defined subjectively by each respondent. Finally, there is an assumption that experience leads to increased understanding of change processes in psychotherapy.

## Conclusion

Psychotherapists are confronted with an overwhelming number of theoretical models and evidence-based treatments from which to choose to guide their practice. Meta-analytic studies show there is relative efficacy among the choices, making the decision of which to use even more difficult. Research showing pantheoretical elements found in all effective models, called the common factors, can offer psychotherapists and educators a focusing point to maximize their effectiveness, regardless of their chosen approach.

In practice, most psychotherapists are trained in, and start practicing from, a traditional theoretical orientation (e.g., psychodynamic, cognitive behavioral, systemic) but they frequently see their approach evolve over time toward an informal eclecticism, emerging primarily from their practice experience with clients. This exploratory qualitative study asked experienced psychotherapists what they believed were the change agents in psychotherapy, and their reports illustrated they had indeed unintentionally gravitated toward eclecticism and a heavy use of common factors—seeing the therapeutic relationship, therapist genuineness, good therapist—client matching, and the role of the client’s efforts toward change, as central components of change in psychotherapy.

Given these findings, an avenue for further inquiry is opened—if psychotherapists are going to eventually evolve in an unplanned eclectic direction guided by their client interactions, are they also concurrently drawn to the common factors? If the answer proves to be yes, why so, and what are the implications for early training? Should the gradual emphasis toward common factors be supplanted with a more intentional focus on them as primary change agents with students and early career clinicians? It could save them time.

Given the wide variety of models available to conduct psychotherapy, more research and training are needed to make psychotherapists aware of the of panthoretical common factors—given they are well supported by research and preferred by clients. Beginning therapists will still likely need a pure-form model at the outset of their career as a way of grounding their practice, but we know it will be supplemented over time by varied skills & techniques from outside their original orientation. Even if a therapist starts their training using an integrative model, we can expect that they will modify it in their own unique way over time as they become mid-career and experienced clinicians. Awareness of the common factors can be validating for clinicians if they are naturally going to gravitate toward using them anyway as their career progresses. It may also avoid them feeling they are unhelpfully deviating from theoretical or evidence-based practice. Perhaps if clinicians begin to intentionally deploy common factors early in their career, it will be more efficient than having their clients teach it to them over time.

## Data Availability

The datasets generated and analyzed during the current study are available from the corresponding author upon reasonable request.

## Appendix

### Experienced therapists’ conceptualizations of the change factors in psychotherapy interview guide

This appendix lists the prompts used to conduct interviews with respondents.
